# Budgetary impact analysis of a primary care-based hepatitis C treatment program: Effects of 340B Drug Pricing Program

**DOI:** 10.1371/journal.pone.0213745

**Published:** 2019-03-14

**Authors:** Eric A. Jones, Benjamin P. Linas, Ve Truong, James F. Burgess, Karen E. Lasser

**Affiliations:** 1 Boston University, School of Public Health, Boston, MA, United States of America; 2 Boston University, School of Medicine, Boston, MA, United States of America; 3 Boston Medical Center, Section of General Internal Medicine, Boston, MA, United States of America; Centers for Disease Control and Prevention, UNITED STATES

## Abstract

**Purpose:**

Safety-net health systems, which serve a disproportionate share of patients at high risk for hepatitis C virus (HCV) infection, may use revenue generated by the federal drug discount pricing program, known as 340B, to support multidisciplinary care. Budgetary impacts of repealing the drug-pricing program are unknown. Our objective was to conduct a budgetary impact analysis of a multidisciplinary primary care-based HCV treatment program, with and without 340B support.

**Methods:**

We conducted a budgetary impact analysis from the perspective of a large safety-net medical center in Boston, Massachusetts. Participants included 302 HCV-infected patients (mean age 45, 75% male, 53% white, 77% Medicaid) referred to the primary care-based HCV treatment program from 2015–2016. Main measures included costs and revenues associated with the treatment program. Our main outcomes were net cost with and without 340B Drug Pricing support.

**Results:**

Total program costs were $942,770, while revenues totaled $1.2 million. With the 340B Drug Pricing Program the hospital received a net revenue of $930 per patient referred to the HCV treatment program. In the absence of the 340B program, the hospital would lose $370 per patient referred. Ninety-seven percent (68/70) of patients who initiated treatment in the program achieved a sustained virologic response (SVR) at a net cost of $4,150 each, among this patient subset.

**Conclusions:**

The 340B Drug Pricing Program enabled a safety-net hospital to deliver effective primary care-based HCV treatment using a multidisciplinary care team. Efforts to sustain the 340B program could enable dissemination of similar HCV treatment models elsewhere.

## Introduction

Hepatitis C virus (HCV) infection is a leading cause of morbidity and mortality in the United States [US], with prevalence estimates between 1–2% or 2.7–3.9 million individuals infected,[1] and in excess of 10,000 deaths annually.[2] Untreated, HCV infection can produce complications including cirrhosis, liver disease, and necessity for liver transplantation,[3] at significant cost.[4]

Historically, gastroenterology or infectious diseases sub-specialty practices have treated HCV. Emergence of directly acting, oral agents with improved efficacy and little toxicity enables HCV treatment in primary care settings.[5] Treating HCV in primary care is important for many reasons. Firstly, sub-specialty treatment capacity is limited in the US [6] and as a result, specialty practices may not be able to meet future demand for HCV treatment. Secondly, referral from primary to sub-specialty care is a well-documented point at which patients are lost to follow-up.[7] Treatment in primary care may offer an opportunity to addrsess this loss to follow-up among difficult to treat patients.

Another impediment to HCV treatment is cost.[8] In 2013, total estimated costs associated with HCV treatment were $6.5 billion.[9] In an effort to combat drug price increases for Medicaid participants, in 1992, the US Congress created the 340B Drug Pricing Program as part of the Public Health Service Act.[10] Under this program, manufacturers provide medications at significant discounts to 340B covered entities. Organizations are able to dispense these discounted medicines, while Medicaid and private insurers reimburse at full-market price. The 340B program can therefore generate net positive returns, which programs can reinvest in care delivery systems in an effort to improve outcomes.[11] Recently, intense policy debate has questioned expansion of the 340B program under the Affordable Care Act, its broader role and suitable application of 340B regulations.[12] Thus, the outlook for the 340B program is uncertain.

Providing HCV treatment in primary care is resource-intensive, requiring significant staff support.[13] Uncertainty surrounding the future of 340B generates challenges for healthcare providers considering implementing primary care-based HCV treatment, as potential cost and revenue remain unclear. Therefore, our objective was to conduct a budgetary impact analysis of a primary care-based HCV treatment program from the perspective of a large safety-net medical center,[13] both with and without 340B program support.

## Methods

We performed a budgetary impact analysis of the Boston Medical Center HCV Primary Care Treatment Program. Assuming the cost perspective of the medical center, we followed methodological guidelines recommended by Mauskopf et al [14] to analyze resource utilization and cost data for 302 patients referred to the program during 2015–2016. Our study involved analyses of retrospective, de-identified data and posed no more than minimal risk to subjects. The Institutional Review Board at Boston University School of Medicine approved the study protocol and did not require collection of written informed consent from participants.

### Population, setting and program description

The Boston Medical Center HCV Primary Care Treatment Program is based in the Adult Primary Care (General Internal Medicine) Practice, an accredited patient-centered medical home situated in New England’s largest safety-net hospital. Program details have been described elsewhere.[13] A multidisciplinary team, including a public health social worker (“case manager”), seven general internists trained to treat HCV (“HCV MD treater”), a pharmacy technician, and a pharmacist staffed the HCV treatment program. The program receives referrals from general internal medicine primary care providers (PCPs); the laboratory via automatic notifications of positive HCV antibody tests; electronic health record (EHR) reports; and peer referrals.

The case manager performs many patient navigation functions (e.g. addresses insurance and transportation barriers, and connects patients to additional services), schedules and provides appointment reminders and telephone support from patient referral to discharge. The case manager provides these services according to patients’ specific barriers to engagement in HCV treatment.

HCV MD treaters assess patient readiness for treatment, and appropriateness of the primary care setting for treatment, perform liver staging, and determine course of treatment. The program has a dedicated pharmacy technician who manages prior authorizations for medications. During visits with the staff pharmacist, patients are provided education about medications and strategies to promote adherence, screened for medication side effects, and administered monitoring laboratory tests. Three months after the end of treatment, the HCV MD treater evaluates presence of HCV RNA to assess for sustained virologic response (SVR; the measure of HCV cure)[15] and counsels patients about reducing risk of reinfection.

### Cost inputs

We identified four components of cost from the medical center perspective: 1) program staff, 2) medications, 3) laboratory tests, and 4) overhead. We excluded costs related to laboratory tests because that infrastructure already exists at our center and the marginal cost of an additional test is trivial relative to costs of program staff and medications.[16] Similarly, we did not explicitly cost space and overhead, because the clinical space utilized by the program is already being used for the general internal medicine practice. The cost of using the space, therefore, is effectively zero, as it is simply the opportunity cost of using the space for delivery of HCV visits rather than primary care visits for the same patient population.

### Program staff

We obtained costs related to salary support for the time spent on HCV care for each team member. Because HCV MD treaters are also primary care providers in the practice and spend much of their time caring for patients who do not have HCV, we estimated the proportion of the total full-time equivalent (FTE) that each provider spent on HCV care. We divided total relative value units (RVUs) generated from evaluating HCV patients by total RVUs generated over the study period. We estimated annual salaries of HCV MD treaters using the Association of American Medical Colleges report of the national median salary among general internal medicine Assistant Professors in the US. We used the real-world annual salary packages for non-MD program staff and included fringe benefit rates. To estimate personnel cost per patient evaluated, we assumed full program capacity and divided the total cost of personnel by the total number of patients served. Finally, we estimated costs (based on national median salary data) to support the salaries of the transient elastography (Fibroscan) technician and gastroenterologist for performing and reading Fibroscans, respectively, and the ultrasound technician and radiologist for performing and reading abdominal ultrasounds, respectively. We only included effort needed to perform and read Fibroscans and ultrasounds completed for the 302 patients included in this analysis.

### Medications

We obtained actual data on the number, type and costs of HCV medications from the hospital-based pharmacy. Boston Medical Center is a disproportionate share safety-net hospital and is thus eligible for the 340B Drug Pricing Program. We obtained the actual 340B “discounted cost” of each medication from the hospital-based pharmacy. Some patients treated in the primary care-based HCV program filled their medications at other pharmacies. We therefore only included medication costs from patients who filled HCV medications directly with the hospital-based pharmacy. A dispensing fee of approximately $10 is passed on to the patient when prescriptions are filled at the hospital pharmacy. However, we decided not to itemize this cost for two reasons: 1) the dispensing fee is built into the price of the medication generally through patient copay, or is directly recovered from the patient’s insurance provider; 2) when compared to the overall cost of HCV meds, we considered the dispensing fee to be trivial and a non-significant source of cost.

### Revenue inputs

In order to assess program revenues, we performed manual chart reviews in the EHR. We collected data regarding payer-specific reimbursement for services delivered and calculated revenue on a per-patient basis. Beginning with the first HCV MD treater visit, in order to estimate total billable services ordered during each encounter, we assumed each patient received a standard list of services over a fixed number of visits. Practice guidelines for HCV management informed this list of services.[15] We adopted this approach in order to 1) provide information regarding pre-treatment intake visits and loss to follow-up and 2) to increase generalizability of our findings to other settings where program uptake may vary, yet a standard set of clinical services is delivered.

We identified three domains of revenue: reimbursement for 1) clinical visits; 2) HCV medications dispensed and 3) laboratory and diagnostic tests, including but not limited to Fibroscans and ultrasounds.

### Reimbursement for clinical services provided

#### Physician visits prior to treatment and three months after treatment completed

Our approach to estimating reimbursement for clinical services provided by the HCV MD treaters was to cost resources consumed at each visit based on the patient’s payer (Medicaid or Medicare). Medicaid or Medicare insured more than 90% of the patients in the program. For the 21 patients who had other types of insurance, we assumed reimbursement to be the weighted average of Medicare and Medicaid reimbursement, using the relative proportion of each payer type within the program as the weight. We stratified by payer type to account for differential reimbursement strategies of Medicare (fee-for-service) and Medicaid (capitated). We collected information regarding reimbursement rates for each payer type and applied the appropriate dollar amounts for covered services on a per-patient basis. Medicare reimbursement rates included a payment of $122 for each level 4 patient intake visit and $75 for each of two level 3 visits with an HCV MD treater. In addition to this payment, Medicare provided reimbursement for all billable services, including labs and imaging, completed during the clinic visit. Medicaid reimbursed at a flat rate of $255 for each HCV MD level 4 patient intake visit regardless of services delivered. Subsequent level-3 encounters with an HCV treater were reimbursed at a flat rate of $54 per visit.

### Pharmacist visits on treatment

We estimated a total of three pharmacy visits over the course of treatment; the pharmacist billed each visit at $90 for Medicare patients and $254 for Medicaid patients.

### Medication reimbursements

The hospital pharmacy provided actual estimates of revenue generated from the 340B program. We only included medication revenue from patients who filled HCV medications at the hospital-based pharmacy. Payers reimbursed the pharmacy at market price; we based estimates of reimbursements using the “Red Book” catalogue of medications.[17]

### 340B Budgetary Impact Analyses

We estimated budgetary impact in the absence of the 340B program, where payers would reimburse HCV medications at the cost of acquisition. As such, the “no 340B” scenario examined increased cost and reduced revenue related to HCV medications, along with cost and revenue related to provision of clinical care.

## Results

### Patient demographic and clinical characteristics

Among the 302 patients referred to the program, three-fourths were male, 53% were white, and the mean age was 45 years. Medicaid and Medicare patients comprised most of the observed payer mix, with Medicaid patients accounting for 77%, and Medicare 15%. (Table 1).

**Table 1:** Demographic and clinical characteristics of patients evaluated by the primary care HCV treatment program at boston medical center, 2015–2016.

Characteristics	Total (n = 302) No. (%)
**Demographic Characteristics**	
Age, mean (SD), y	44.6 (13.0)
Male	228 (75.5)
Race	
African American	86 (28.5)
Caucasian	160 (53.0)
Other	7 (2.3)
Decline or Missing	49 (16.2)
Insurance	
Medicaid	234 (77.5)
Medicare	47 (15.5)
Private	16 (5.3)
Other	5 (1.7)
**Clinical Characteristics**	
Liver Stage^a^	
F0	19 (6.3)
F1	32 (10.6)
F2	26 (8.3)
F3	18 (6.0)
F4	11 (3.6)
Staging not completed	197 (65.2)
Genotype	
1	163 (54.0)
1/2	1 (0.3)
1/3	2 (0.7)
2	20 (6.6)
3	36 (11.3)
3/4	1 (0.3)
4	8 (2.7)
Not completed/ Undetectable viral load	73 (24.2)

^a^Liver staging by transient elastography (Fibroscan).

**Fig 1:**
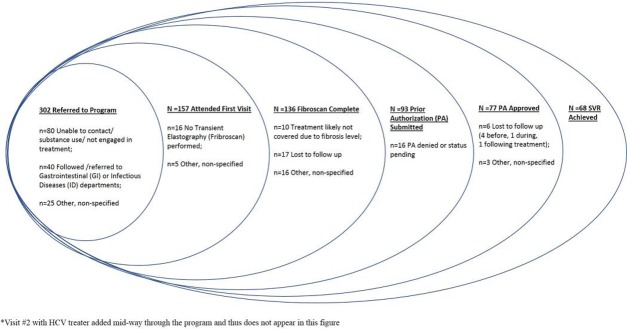
Primacy care HCV treatment program cascade of care.

One hundred fifty-seven of the 302 patients (52%) referred for treatment attended an initial HCV MD treater visit (Fig 1). One hundred and forty-five patients (48%) did not engage in primary care treatment, were already receiving specialty care, or were referred to specialty care. Of the 157 patients who attended the initial visit, 136 (87%) completed a Fibroscan for liver staging. Among these 136 patients, the pharmacy technician submitted a prior authorization (PA) request for 93 (68%). Of these 93 patients, insurance companies approved treatment for 77 (83%), and 70 of these 77 patients (91%) intimated treatment. All 68 patients (97%) who attended a visit three months after completing treatment achieved SVR.

### Economic evaluation of the HCV treatment program cost

We estimated the seven HCV MD treaters contributed a total of 0.18 FTE to the program. Based on a national median salary of $153,000, HCV MD treater salaries accounted for $27,000 of cost. Costs attributable to salary support for program staff included $120,000 for a full-time pharmacist, $60,000 each for a full-time pharmacy technician, and full-time case manager. Costs for other staff members included $4,860 for 1.3% FTE radiologist; $4,940 for 1.3% FTE gastroenterologist; $2,270 for 6.3% FTE ultrasound technician; and $3,700 for 3.3% FTE Fibroscan technician. In total, we estimated salary costs at $282,770 annually, or approximately $940 per patient referred to the program, and $4,160 per patient who achieved SVR. The cost of medications dispensed to patients totaled approximately $660,000 (Table 2).

**Table 2:** Total costs of the primary care HCV treatment program, related to staffing and wholesale acquisition cost of HCV medications, 2015–2016.

**Program staff salary**^**a**^^,^^b^^,^^c^	
0.18 full-time equivalent (FTE) physicians (divided among 7 MDs)	$27,000
1.0 FTE Pharmacist	$120,000
1.0 FTE Pharmacy technician	$60,000
1.0 FTE Case Manager (public health social worker)	$60,000
0.013 FTE Radiologist	$4,860
0.063 FTE Ultrasound technician	$2,270
0.033 FTE Fibroscan technicians	$3,700
0.013 FTE Gastroenterologist	$4,940
**Total staffing costs**	$282,770
**Most frequently prescribed therapeutic agents**	
Daclatasvir^d^	$120,700
Ledipasvir and Sofosbuvir^d^	$226,300
Sofosbuvir^d^	$98,000
Additional HCV medications^e^	$215,000
**Total costs of all prescribed medications**	$660,000
**Total program costs**	$942,770

^a^Annual salaries of physicians from the Association of American Medical Colleges; based on the national median salary among General Internal Medicine, Radiology, and Gastroenterology Assistant Professors in the United States.

^b^Annual salaries for the pharmacist, pharmacy technician and case manager were obtained directly from the program leadership.

^c^Annual salaries for the Ultrasound technician and Fibroscan technician were based upon median salaries obtained from Bureau of Labor and Statistics.

^d^Value represents wholesale acquisition cost. $20,115.90 for Daclatasvir (Daklinza), $30,173.85 for Ledipasvir and Sofosbuvir, and $26,821.20 for Sofosbuvir.

^e^Other HCV medications which were prescribed include Simeprevir, peg interferon alfa-2a, Copegus/Rebetol/Ribasphere, Ombitasvir/Paritaprevir/Ritonavir, Paritaprevir/Ritonavir /Dasabuvir, and Elbasvir/Grazoprevir.

### Revenue

#### 340B Pharmacy revenue

Nearly half (28/68) of patients who received treatment in the program filled prescriptions at the hospital-based pharmacy. Reimbursement for these medications accounted for the largest portion of program revenue, at approximately $1.1 million (Table 3), resulting in a net revenue of approximately $440,000.

**Table 3:** Total revenue of the primary care HCV treatment program, related to reimbursement for medications and billable services, 2015–2016.

**Reimbursement for all HCV medications**^**a**^ **with 340B program**	$1.1M
**Provider services delivered for HCV diagnosis & clinical management**^b^	
Medicaid	$27,800
Medicare	$27,600
Total	$55,400
**Clinic visits with HCV MD treater**^c^	
Medicaid	$7890
Medicare	$5880
Total	$13,770
**Visits with pharmacist**^d^	
Medicaid	$36,570
Medicare	$2,700
Total	$39,270
**Total revenue of services provided**	$108,440
**Total revenue**	$1.22M

^a^Value represents total revenue for all HCV medications dispensed directly to patients through the safety-net hospital pharmacy.

bClinical services include billing for outpatient visits where services performed, but not limited to, consist of: HCV genotyping and viral load, Fibroscan, ultrasound, complete blood count, and metabolic panel.

^c^HCV MD treater visits include those in addition to the initial intake visit upon referral. These level 3 encounters include visits to review liver staging data, additional clinical management, and one visit to ascertain whether patient achieved sustained virologic response (SVR) three months following treatment completion.

^d^Visits with pharmacist to discuss medication regimen, importance of adherence, adverse events, etc. These visits are billed separately from HCV treater visits.

#### Provider services

HCV evaluation and treatment produced revenue of $1,600.00 per Medicare and $1,380.00 per Medicaid patient evaluated.

The total cost of the program was $942,770 (Table 2), with revenue estimated at approximately $1.2 million (Table 3). Based on the total revenue and number of patients evaluated, we estimate the program generated net revenue of $930 per patient referred to the program (Table 4).

**Table 4:** Total Costs and revenue of primary care HCV treatment program, according to presence or absence of the 340b drug pricing program.

Component	With 340B		Without 340B	
	Cost	Revenue	Cost	Revenue^a^
**Billable Services**^**b**^	-	$63,400	-	$63,400
**HCV MD Treater**^**c**^	$42,770	$5,770	$42,770	$5,770
**Case Manager**	$60,000	$9820	$60,000	$9820
**Pharmacist and pharmacy technician**^**d**^	$180,000	$29,450	$180,000	$29,450
**HCV Medications**^**e**^	$660,000	$1.12M	$1.24M	$1.3M
**Total**	$942,770	$1.22M	$1.50M	1.41M
**Net**	$282,000		(-$112,270)	
**Net per patient**	$930		(-$370)	

^a^Revenue for billable services, HCV MD treater & pharmacy support is not affected by the presence or absence of the 340B drug pricing program.

^b^Billable services include all procedures and care related to clinical HCV management (e.g. Fibroscan, genotyping, etc.) and reimbursement for the clinic visit at which these services were delivered.

^c^HCV MD treater costs includes total salary support for all program MDs and technicians. Revenue generated was from reimbursement for clinic visits over the course of treatment.

^d^Pharmacy costs includes salary support for pharmacist, pharmacy technician. Revenue includes reimbursement for billable services related to pharmacist activities with program patients.

^e^Cost and revenue only considered for medications dispensed by the safety-net pharmacy directly to patients.

#### 340B budgetary impact analysis

Without the 340B benefit, we estimate a net cost of $370 per patient referred to the HCV treatment program. This net reduction is largely due to the fact that, in the absence of 340B specialty pricing, costs of medications rose to just over $1.2 million (Table 4), leading to net returns of just over $62,000, or an overall reduction of roughly $380,000.

## Discussion

The 340B program enabled a safety-net hospital to deliver primary care-based HCV treatment using a multidisciplinary care team, and resulted in a net revenue of $930 per patient referred. Without the 340B program, the practice would experience a net loss of $370 per patient referred and would likely not be sustainable in resource-poor settings, which disproportionately care for HCV-infected patients. Further, if the 340B program were removed, patient adherence to treatment could decline, as the hospital might be unable to support the ancillary services provided by the case manager and pharmacist that facilitate adherence to treatment.

The hospital invested 340B revenue to support case management, including providing case management and navigation services to HCV-infected patients in settings outside of GIM, as well as the general operating expenses of the safety-net hospital. Case management is of particular import, as patients at greatest risk for HCV infection often have comorbid substance use [18] and/or mental health disorders,[19] and are more likely to receive their care at safety-net hospitals.[20] Such patients may derive the greatest benefit from services of a case manager who may identify and address barriers to engagement and retention in HCV treatment. We are unaware of other funding mechanisms that could support a multidisciplinary HCV treatment program such as the one described in this study. Further, the costs associated with these ancillary services may be too great for safety-net providers to subsidize long-term, highlighting the importance of financial support from the 340B program.

Given the projected increase in HCV-related disease, HCV infection will likely remain a significant burden to the US health care system.[21] Benchmarks for HCV treatment proposed by the Institute of Medicine and Centers for Disease Control may be difficult to attain given the limited capacity of safety-net providers to treat HCV in specialty settings.[22] Expansion of HCV treatment into primary care is an ideal alternative and can be effectively delivered [23] in a cost-effective manner[24–28].

Staffing costs in the model were substantial. Salary support for a case manager, pharmacy technician, and pharmacist may be an impediment to smaller or community-based practices seeking to replicate this program. Despite these costs, the multidisciplinary nature of the program staff is likely essential to its success. Similarly, smaller practices may lack access to a Fibroscan machine and may need to rely on blood testing (e.g. Fibrosure) for liver staging.

Treatment of HCV in primary care likely requires significant up-front investment, which can be a barrier for resource limited health care organizations. Consequences of failure to treat HCV are stark. These consequences include continued transmission,[29] re-infection,[30] and long-term health effects such as cirrhosis and end-stage liver disease, for which early treatment may be a cost-effective strategy.[31, 32]

Our study has several limitations. First, it was conducted at a single site so may lack generalizability. Massachusetts has among the most generous Medicaid coverage for HCV treatment and does not restrict treatment to advanced fibrosis or specialty settings. Secondly, we assumed a fixed number of visits per patient evaluated in the program; it is possible that some patients required additional visits. We also assume fixed costs for medicatons. Should drug costs change significantly, additional downstream effects on program costs, revenue and patient uptake are possible. Additionally, we have not included costs related to space, and capital equipment, such as a Fibroscan machine, which may be of significant import to smaller practices. We did not consider costs from the patient perspective; the impact of such costs, though not directly related to the practice budget, warrants further investigation. Finally, insurers such as Medicaid may directly negotiate medication pricing with pharmaceutical companies and therefore the 340B drug discount program may not be applicable.

We are unaware of previous studies which have examined budgetary impacts of 340B pricing on a multidisciplinary HCV care delivery model. Previous studies such as The Extension for Community Healthcare Outcomes (ECHO) project demonstrated clinical and cost-effectiveness of HCV care delivery in primary care;[23] however, the effect of 340B pricing was not examined.

Expansion of treatment into primary care will likely be necessary to address the expected rise in incidence of HCV infection. At the same time, treatment models like those we describe could also be implemented into existing specialty HCV practices. Implementation and sustainment of multidisciplinary treatment models similar to the program evaluated here will likely depend upon maintenance of the 340B program. Future policy discussions should include consideration of ways to preserve the current 340B program or, alternatively, creation of funding mechanisms that assist resource-limited providers with care delivery to vulnerable populations at increased risk for HCV infection.
